# Predicted HIV-1 coreceptor usage among Kenya patients shows a high tendency for subtype d to be cxcr4 tropic

**DOI:** 10.1186/1742-6405-9-22

**Published:** 2012-07-28

**Authors:** Veronica Wambui, Michael Kiptoo, Joyceline Kinyua, Irene Odera, Edward Muge, Peter Muiruri, Raphael Lihana, Peter Kinyanjui, Elijah M Songok

**Affiliations:** 1 Department of Biochemistry, University of Nairobi, Nairobi, 30197-00100, Kenya; 2 Centre for Virus Research, Kenya Medical Research Institute, Nairobi, 54848-00200, Kenya; 3 Comprehensive Care Centre, Kenyatta National Hospital, Nairobi, 2146-00202, Kenya

**Keywords:** Co-receptor usage, HIV-1, Sub-type D

## Abstract

**Background:**

CCR5 antagonists have clinically been approved for prevention or treatment of HIV/AIDS. Countries in Sub-Saharan Africa with the highest burden of HIV/AIDS are due to adopt these regimens. However, HIV-1 can also use CXCR4 as a co-receptor. There is hence an urgent need to map out cellular tropism of a country’s circulating HIV strains to guide the impending use of CCR5 antagonists.

**Objectives:**

To determine HIV-1 coreceptor usage among patients attending a comprehensive care centre in Nairobi, Kenya.

**Methods:**

Blood samples were obtained from HIV infected patients attending the comprehensive care centre, Kenyatta National Hospital in years 2008 and 2009. The samples were separated into plasma and peripheral blood mononuclear cells (PBMCs). Proviral DNA was extracted from PBMCs and Polymerase Chain reaction (PCR) done to amplify the HIV *env* fragment spanning the C2-V3 region. The resultant fragment was directly sequenced on an automated sequencer (ABI, 3100). Co-receptor prediction of the *env* sequences was done using Geno2pheno [co-receptor], and phylogenetic relationships determined using CLUSTALW and Neighbor Joining method.

**Results:**

A total of 67 samples (46 treatment experienced and 21 treatment naive) were successfully amplified and sequenced. Forty nine (73%) sequences showed a prediction for R5 tropism while 18(27%) were X4 tropic. Phylogenetic analysis showed that 46(69%) were subtype A, 11(16%) subtype C, and 10(15%) subtype D. No statistical significant associations were observed between cell tropism and CD4+ status, patient gender, age, or treatment option. There was a tendency for more X4 tropic strains being in the treatment experienced group than the naive group: Of 46 treatment experiencing participants, 14(30%) harboured X4, compared with 4(19%) of 21 of the treatment-naïve participants, the association is however not statistically significant (p = 0.31). However, a strong association was observed between subtype D and CXCR4 co- receptor usage (p = 0.015) with 6(60%) of the 10 subtype D being X4 tropic and 4(40%) R5 tropic.

**Conclusion:**

HIV-1 R5 tropic strains were the most prevalent in the study population and HIV infected patients in Kenya may benefit from CCR5 antagonists. However, there is need for caution where subtype D infection is suspected or where antiretroviral salvage therapy is indicated.

## Background

Human immunodeficiency virus (HIV) is the causes of acquired immunodeficiency syndrome (AIDS) [1]. Human immunodeficiency virus has currently surpassed malaria as a leading cause of adult infectious disease mortality worldwide [2]. The Kenya AIDS indicator survey (KAIS), reported by the National AIDS/STIS Control Programme (NASCOP) found that 7.1% of adults (aged 15-64 years) in Kenya were infected with HIV by end of 2007 [3]. HIV is dependent on a host cell for its replication, and requires binding to receptors on the cell surface in order to gain entry. The first receptor is CD4, which is the main receptor and is always the same for each virus particle. There are other two receptors CCR5 and CXCR4 that serve as co-receptors [4]. The co-receptors are used by HIV to infect specific cell types; this phenomenon is referred to as HIV tropism. Non-syncitia-inducing (NSI) strains use the betachemokine receptor, CCR5, for entry and thus able to replicate in macrophages and CD4 positive T-cells. Syncitia inducing (SI) strains replicate in CD4 positive T-cells and use the alpha chemokine receptor, CXCR4 [5]. The preferred phenotypic designations are R5 for the non-syncytium inducing CCR5 using HIV and X4 for the syncytium inducing CXCR4 using HIV [6]. R5 isolates are present early after seroconversion indicating their role in initiation of HIV-1 infection. The eventual use of CXCR4 by X4 isolates of HIV-1 which infect primary T cells and T cell lines closely correspond to the onset of AIDs, although R5 isolates do persist throughout the entire course of infection [6-8]. HIV-1 co-receptor usage has been reported to be fairly stable after HAART therapy [9]. Some ART drugs can lead to suppression of CXCR4 strains of HIV [10]. Of the five HIV-1 gp120 hyper-variable domains, V3 has been a target of interest for entry-based inhibitors because of its critical role in defining the specificity of HIV-1 envelope interaction with cellular co-receptor molecules, usually, CCR5 or CXCR4, to facilitate entry into target cells. It encodes the key determinants of viral co receptor usage [11]. Recombinant virus phenotypic entry assays are considered as the gold standard method for determining co-receptor usage [12]. However, their routine use is hampered by technical and cost limitations. Of late the *in silico* approaches are gaining popularity given the simplicity of this strategy and the fact that *env* sequences are increasingly becoming available globally. These include among others, Geno2pheno [co-receptor] which predicts whether the corresponding virus is capable of using CXCR4 or CCR5 as a co receptor [13,14].

By the end of 2007, only 177,000 (40%) of the estimated 470,000 people in need of ART were receiving treatment in Kenya [15]. In Kenya, the first line regimen consists of two nucleoside reverse transcriptase inhibitors (NRTIs) and a non-nucleoside reverse transcriptase inhibitor (NNRTI)/Ritonavir boosted protein inhibitor (PI/r). While the recommended second line regimen consists a fixed drug combination of Didanosine (ddI)/Tenofvir (TDF), Abacavir (ABC) and Lopinavir/ritonavir (LPV/r) [16].

With the introduction of the CCR antagonists for HIV therapy, there is a need to map out the cellular tropism of circulating HIV-1 strains in Kenya. The HIV-1 subtype diversity in Kenya may have an influence on how the CCR5 antagonists are used in Kenya following studies done in Uganda that have demonstrated a high tendency for subtype D to be CXCR4 [17,18]. We therefore carried out a preliminary analysis to determine co-receptor usage in HIV-infected patients attending an outpatient clinic at a tertiary hospital in Nairobi, Kenya. Furthermore, we aimed to evaluate if a correlation exists between HIV-1 tropism, HIV-1 subtypes, and current antiretroviral treatment strategies in Kenya.

## Methods

### Study population

This was a cross sectional study. The population consisting of antiretroviral therapy experienced patients and treatment naive patients were recruited from the Comprehensive.

Care Centre, Kenyatta National Hospital in 2008 and 2009. The fixed dose combinations for the treatment group were: Zidovudine (AZT)/Stavudine (d4T) + Lamivudine (3TC) + Nevirapine (NVP)/Efavirenz (EFV) and Tenofovir Disoproxil Fumarate (TDF)/Abacavir (ABC) + 3TC/Didanosine (ddI) + Liponavir/Ritonavir (LPV/r*). The blood from all the subjects was collected for CD4+ count detection and the peripheral blood mononuclear cells (PBMCs) for isolating HIV-1 strains. All subjects signed informed consent forms before blood collection. This study was approved by the Kenya medical Research Institute Scientific Steering Committee and Ethical Review Board (Ref. KEMRI SSC No. 1252).

### CD + T cell counts and PBMC extraction

CD4+ T cell counts of peripheral blood were determined using FACSCOUNT (Becton-Dickinson, Beiersdorf, Germany). Peripheral blood mononuclear cells were extracted from whole blood by density gradient centrifugation and stored at −30°C.

### Extraction and amplification of proviral HIV DNA

Samples were archived, thawed and proviral DNA extracted using Gibco BRL kit as per Manufacturers instructions. A part of the HIV-1 group M env gene covering the C2V3 region (corresponding to 6975–7520 nt in HIV-1 HXB2) was amplified by nested polymerase chain reaction (PCR) with primers M5(5’-CCCCTATTCCTTTTCCCCTTCTTTTAAAA-3’) and M10(5’- CCAATTCCCATACATTATTGTGCCCCAGCTGG-3’) in the first round and M3(5’- GTCAGCAACAGTACAATGACACATGG-3’) and M8(5’- TCCTTCCATGGGAGGGGACTACATTGC-3’) in the second round according to manufacturers instructions. Amplification was done with one cycle of 10 min at 95°C, 35 cycles of 30s at 95°C, 30s at 55°C and 1 min at 72°C followed by a final extension of 10 min at 72°C. PCR amplification was confirmed by visualization with ethidium bromide staining of the gel.

### Sequencing and subtyping of the C2V3 env region

The resultant 550 bp fragment was sequenced using an automated ABI 3100 sequencer. Sample nucleotide sequences were aligned with HIV-1 subtype/circulating recombinant form (CRF) reference sequences from the Los Almos database using CLUSTAL W with minor manual adjustments. A phylogenetic tree was constructed by the neighbour joining method and its reliability was estimated by 1000 bootstrap replications. The profile of the tree was visualized with Tree View PPC version 1.6.5. To improve the accuracy of subtyping, we used the NCBI Viral Genotyping tools (http://www.ncbi.nlm.nih.gov/projects/genotyping/formpage.cgi) and REGA subtyping tools v2.0 (http://www.bioafrica.net/rega-genotype/html/subtypinghiv.html). The C2-V3 region nucleotide sequences (550 bp) were translated into the corresponding 35 amino acids using Genetic Information Processing Software (Genetyx-Win) version 4.0 (Genetyx, Tokyo, Japan). Sequences with premature stop codons were excluded from further analyses.

### Co-receptor usage prediction

Geno2pheno [co-receptor] tool (with a false positive rate of 10%) was used to predict HIV-1 co receptor usage. (http://coreceptor.bioinf.mpi-inf.mpg.de/) was used. Geno2pheno is available at http://co receptor.bioinf.mpi-inf.mpg.de/cgi-bin/co receptor.pl (September 2010). Subtype D genotypic algorithm based on 11/25 and a net charge rules was further used to predict tropism of the sub-type D sequences. One of the following criteria was required for predicting subtype D CXCR4 co-receptor usage: (i) R/K at position 11 of V3; (ii) R at position 25 of V3 and a net charge of ≥ +5; (iii) a net charge of ≥ +6 [14].

### Statistical analysis

Correlations of HIV-1 subtype, ART regimen, gender, CD4 count and viral tropism were performed by Chi square test using SPSS v.16 software (IBM Company, New York). P values less than 0.05 were considered statistically significant.

## Results

### Clinical and general characterization of study population

A total of 67 samples were successfully amplified and sequenced. Of these, 46 individuals were from the ART experienced group while 21 individuals were from the treatment naive group. The mean age of the study population was 39(16–65 years), 23(34%) were male and 44(66%) were female. The mean CD4 count of the treatment experienced group was 249 cells/mm^3^ of whole blood and 375 cells/mm^3^ of whole blood for the treatment naive group. Twenty two (48%) of the treatment experiencing group were on AZT/d4T + 3TC + NVP/EFV fixed dose combination while 24(52%) were on TDF/ABC + 3TC/ddI + LPV/r* fixed dose combination.

### HIV-1 subtypes

**Figure 1:**
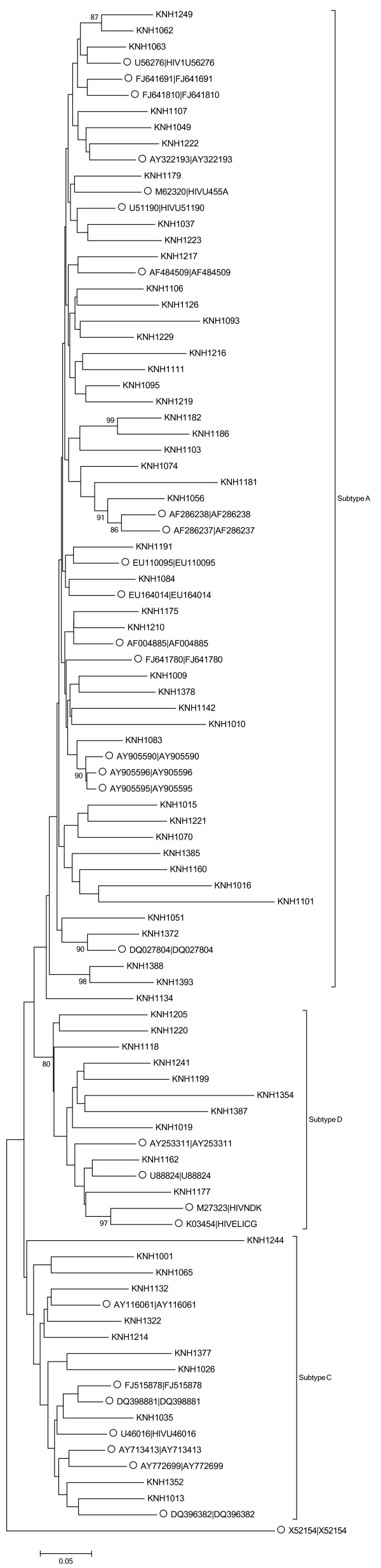
**Phylogenetic tree of HIV-1 env-C2V3 region.** C2V3 region sequences of the study population were aligned and compared with reference sequences obtained from the Los Alamos HIV database. Phylogenetic relationships were constructed by neighbor-joining method and rooted with X52154. The bootstrap values (of 1000 replicates) above 70% are indicated next to the node. Brackets on the right indicate the subtype clusters while the labelled branches indicate the reference sequences.

Of the total 67 samples successfully amplified and sequenced, 46(69%) were subtype A 11(16%) subtype C and 10(15%) subtype D (Figure 1).

### Predicted co-receptor usage

Of the 67 samples successfully amplified, sequenced and their C2-V3 region 550 bp fragment translated to corresponding 35 amino acids, 49(73%) were predicted to be R5-tropic stains while 18(27%) were predicted to be X4-tropic strains using geno2pheno [co-receptor] bioinformatics tool. There was no discrepancy in tropism prediction for subtype D sequences using Geno2Pheno [co-receptor] and subtype D genotypic algorithm (Table 1). There was a tendency of a higher number of X4 tropic viruses being in the treatment experienced group than the naive group: Of 46 treatment experiencing participants, 14(30%) harboured X4, compared with 4(19%) of 21 of the treatment-naïve participants, the association is however not statistically significant (p = 0.31). A strong association was however observed between subtype D and CXCR4 co-receptor usage (p = 0.015) (Table 2). There was no association between co-receptor usage and CD4 count, gender and age of the study participants (p = 0.26), (p = 0.97) and (p = 0.22) respectively (Table 2).

**Table 1:** Subtype D sequences tropism prediction by Geno2pheno tool and subtype D genotypic algorithm

**Patient ID**	**Subtype D genotypic algorithm**				**Geno2Pheno tool**
	**Amino acid at position 11**	**Amino acid at position 25**	**Net charge**	**Strain**	
KNH1220	G	K	6	X4	X4
KNH1118	S	D	4	R5	R5
KNH1019	R	N	9	X4	X4
KNH1177	R	K	6	X4	X4
KNH1162	S	K	6	X4	X4
KNH1387	G	N	5	R5	R5
KNH1354	K	K	4	X4	X4
KNH1076	S	R	11	X4	X4
KNH1109	S	D	5	R5	R5
KNH1241	S	T	5	R5	R5

**Table 2:** HIV-1 co-receptor usage and its associated influence factors

	**R5 co-receptor usage**	**X4 co-receptor usage**		
**CD4 + T count (cells/mm**^**3**^**)**				
CD4 < 200	18 (66.7%)	9 (33.3%)	p = 0.26	
200 = CD4 < 350	15 (93.8%)	19 (6.2%)		
350 = CD4 < 500	11 (73.3%)	4 (26.7%)		
CD4 > =500	5 (55.6%)	4 (44.4%)		
**Gender**				
Female	32 (72.7%)	12 (27.3%)	p = 0.97	
Male	17 (73.9%)	6 (26.1%)		
**Age**				
16-25 years	2 (50.0%)	2 (50.0%)	p = 0.22	
26-35 years	15 (83.3%)	3 (16.7%)		
36-45 years	20 (66.7%)	10 (33.3%)		
> 46 years	12 (80.0%)	3 (20.0%)		
**Therapeutic regimen**				
AZT/d4T + 3TC + NVP/EFV	13 (59.1%)	9 (40.9%)	p = 0.31	
TDF/ABC + 3TC + DDI/LPV/r*	19 (79.2%)	5 (20.8%)		
Treatment Naïve	17 (81%)	4 (19%)		
**HIV-1 Subtype**				
A	36 (78.3%)	10 (21.7%)	p = 0.261	p = 0.04
C	9 (81.8%)	2 (18.2%)	p = 0.504	
D	4 (40%)	6 (60%)	p = 0.015	

## Discussion

Despite the impending introduction of CCR5 antagonists as a treatment of option of HIV in Sub-Saharan countries, little is known about HIV co-receptor usage prevalent among infected populations. Our data demonstrates a high prevalence of R5-using strains among patients attending Kenyatta National Hospital HIV/AIDS comprehensive care clinic in Nairobi. This finding is in line with our earlier evaluation of HIV-1 *coreceptor usage* among a sexually transmitted infection clinic attendees and HIV-1 infected infants at a children’s home [19,20]. Our data hence confirms the predominance of R5 strains in HIV infected populations in the country.

Though subtype D constituted only fifteen per cent of the study population, they represented the subtype with highest prevalence of X4 tropic strains. Our observation is in agreement with two other studies done in Uganda. One study aimed to determine whether differences exist in co-receptor use in patients infected with subtypes A & D in a rural Ugandan cohort found the probability of having an X4 virus being higher in subtype D infections than subtype A infections [17]. The second study was conducted among HIV-infected Ugandan infants where tropism assay and phylogenetic methods revealed X4 tropism and subtype D association [18]. Although HIV-1 co-receptor usage and HIV-1 subtype relationship has been well studied in Uganda, less is known about Kenya. Our data therefore confirms the strong relationship between HIV-1 subtype D and X4 infections in Kenya.

HIV-1 subtype D infection is associated with rapid loss of CD4 cells and faster disease progression than any other subtype [21]. Similarly, HIV-1 X4-tropic strains have been confirmed to be associated with faster disease progression compared to HIV-1 R5-tropic strains. Therefore, co-receptor usage could be one explanation for the fast disease progression HIV subtype D strains. In a study aiming to compare the rate of disease progression based on rate of CD4 decline prior to ART, and the initial and subsequent virological response to ART in an ethnically diverse population in south London infected with diverse subtypes, Eastbrook et al. found subtype D to be associated with both a statistically significant four-fold faster rate of CD4 decline and a higher rate of virological rebound on ART compared with subtype A, B, or C. In Uganda, HIV patients infected with subtype D have been shown to have a more severe dementia as compared with those infected with other subtypes [22]. In a follow-up of HIV seropositive women in Daresalam, individuals infected with subtype D experienced the most rapid progression to death. The above associations confirm our findings of subtype D with a higher prevalence of X4 tropic strains.

As use of antiretroviral treatment is now a common feature in HIV/AIDS management, our study also aimed to evaluate if a correlation exists between HIV-1 tropism and currently used therapeutic regimens. The results showed a higher CXCR4 utilization among antiretroviral therapy group than in the treatment naive population, though the difference was not statistically significant. Some groups in developed countries [23] have found a significantly higher prevalence of X4 viruses among antiretroviral treatment groups. We caution however that our methodology did not take into effect the presence of dual or mixed CCR5/CXR4 viral strains which have been found to be prevalent in other antiretroviral experienced populations. The mechanism responsible for the emergence of CXCR4- using viruses on antiretroviral treatment groups remains unclear. Several hypotheses have been forwarded. Either, partially suppressive therapy may lead to an increase in HIV-specific T cell responses [24]. Because X4-tropic variants may be more susceptible to cytotoxic T cell responses than R5-tropic viruses, increases in HIV-specific T cell responses during partial treatment-mediated viral suppression might select against X4-tropic viruses [25], or antiretroviral therapy may reduce CCR5 expression on T cells, presumably as a consequence of reductions in T cell activation [26,27], potentially selecting for X4-tropic viruses [28]. Lastly, certain antiretroviral drugs may preferentially select for one virus population, either because of enhanced activity against X4 viruses as has been suggested for enfuvirtide [29] or because of suboptimal drug metabolism in the cellular reservoirs for X4 viruses as has been suggested for zidovudine [30].

Our study has two potential shortcomings. First, the small sample size for subtype D and subtype C could have led to the cellular tropism not being well documented for these subtypes, though subtype A has been reported to be the predominant subtype in Kenya. Second, we did not confirm the V3 prediction done by genotypic algorithm by phenotypic assay. Genotypic algorithms have been reported to lack sensitivity for predicting CXCR4 usage [31]. There is therefore need to improve their sensitivity. The genotype-based co-receptor predictors should therefore not be used alone in a clinical setting and even if they approached the sensitivity of phenotypic assays, they would need validation to be used as a wide spread clinical tool.

In conclusion, we infer that HIV-1 R5 tropic strains were more prevalent in the study population suggesting the potential benefit of CCR5 antagonists as a therapeutic option in Kenya. However the observed higher prevalence of X4 strains among subtype D, may call for caution in administration of CCR5 antagonists to patients where subtype D infection is suspected and or where salvage antiretroviral therapy is indicated.

### Genebank accession numbers

The sequences from this study has been deposited at the Los Alamos HIV database under accession numbers JN 593245-JN593311.

## Competing interests

The authors declare that they have no competing interests.

## Authors’ contributions

ES and VW conceived the experiment. VW and IO performed the laboratory procedures while PM helped with patient recruitment and clinical follow-up. ES, MK, VW, RL and JK performed bioinformatics and data analysis. VW and ES drafted the manuscript while EM and PK provided scientific guidance. All authors read and approved the final manuscript.

## References

[B1] Weiss RA How does HIV cause AIDS? Science 2003 260 5112 1273 1279 8493571 10.1126/science.8493571

[B2] Nelson Michael L Host genetic influences on HIV-1 pathogenesis Curr Opin Immunol 1999 11 466 474 10448150 10.1016/S0952-7915(99)80078-8

[B3] National AIDS and STI Control Programme Kenya AIDS Indicator Survey 2007 2008 Nairobi, Kenya: Preliminary Report. Ministry of Health

[B4] Berger E Murphy P Farber J Chemokine receptors as HIV-1 coreceptors Annu Rev Immunol 1999 17 657 700 10358771 10.1146/annurev.immunol.17.1.657

[B5] Coakley E Petropoulos CJ Whitcomb JM Assessing chemokine co-receptor usage in HIV Curr Opin Infect Dis 2005 18 1 9 15 15647694 10.1097/00001432-200502000-00003

[B6] Berger EA HIV entry and tropism: the chemokine receptor connection AIDS 1997 11 S3 S16 9451961

[B7] Moore JP Trkola A Dragic T Co-receptors for HIV-1 entry Curr Opin Immunol 1997 9 551 562 9287172 10.1016/s0952-7915(97)80110-0

[B8] Berger EA Doms RA Fenyo EM Korber BTM Littman DR Moore JP Sattentau QJ Schuitemaker H Sodroski J Weiss RA A new classification for HIV-1 Nature 1998 391 240 9440686 10.1038/34571

[B9] Lehmann C Daumer M Boussaad I Sing T Beerenwinkel N Lengauer T Schmeisser N Wyen C Fatkenheuer G Kaiser R Stable co-receptor usage of HIV in patients with ongoing treatment failure on HAART J Clin Virol 2006 37 4 300 304 17005445 10.1016/j.jcv.2006.08.008

[B10] Philpott S Weiser B Anastos K Kitchen CM Robinson E Meyer WA Sacks HS Brunner C Burger H Preferential suppression of CXCR4-specific strains of HIV by antiviral therapy J Clin Invest 2001 107 4 431 438 11181642 10.1172/JCI11526 PMC199259

[B11] Hwang SS Boyle TJ Lyerly HK Cullen BR Identification of the envelope V3 loop as the primary determinant of cell tropism in HIV-1 Science 1991 253 71 74 1905842 10.1126/science.1905842

[B12] Whitcomb J Huang W Fransen S Development and characterization of a novel single-cycle recombinant-virus assay to determine HIV-1 coreceptor tropism Antimicrob Agents Chemother 2007 51 566 575 17116663 10.1128/AAC.00853-06 PMC1797738

[B13] Sing T Low AJ Beerenwinkel N Sander O Cheung PK Domingues FS Buch J Daumer M Kaiser R Lengauer T Harrigan PR Predicting HIV-coreceptor usage based on genetic and clinical covariates Antivir Ther 2007 12 1097 1106 18018768

[B14] Raymond S Delobel P Chaix ML Cazabat M Encinas S Bruel P Sandres-Saune K Marchou B Massip P Izopet J Genotypic prediction of HIV-1 subtype D tropism Retrovirology 2011 8 56 21752271 10.1186/1742-4690-8-56 PMC3146927

[B15] UNAIDS/WHO Epidemiological Fact Sheets on HIV and AIDS-Kenya 2008 http://www.who.int/hiv/pub/epidemiology/pubfacts/en/.

[B16] Ministry Of Health Guidelines for Antiretroviral Therapy Drug Therapy in Kenya Third Nairobi, Kenya: Ministry Of Health

[B17] Kaleebu P Nankya IL Yirrell LD Shafer LA Shafer LA Kyosiimire-Lugemwa Lule BD Morgan D Beddows S Weber J Whitworth JAG Relation between chemokine receptor use, disease stage, and HIV-1 subtypes A and D results from a rural ugandan cohort J Acquir Immune Defic Syndr 2007 45 28 33 17310935 10.1097/QAI.0b013e3180385aa0

[B18] Church DJ Huang W Mwatha A Toma J Stawiski E Donnell D Guay AL Mmiro F Musoke P Jackson BJ Parkin N Eshleman HS HIV-1 tropism and survival in vertically infected Ugandan infants J Infect Dis 2008 197 10 1382 1388 18444795 10.1086/587492

[B19] Lihana RW Khamadi SA Lwembe RM Kinyua JG Muriuki JK Lagat NJ Okoth FA Makokha PE Songok EM HIV-1 subtype and viral tropism determination for evaluating antiretroviral therapy options: an analysis of archived Kenyan blood samples BMC Infect Dis 2009 9 215 20040114 10.1186/1471-2334-9-215 PMC2804586

[B20] Lwembe R Lihana RW Ochieng’ W Panikulam A Mongoina CO Palakudy T De Koning H Ishizaki A Kageyama S Musoke R Owens M Songok EM Okoth FA Ichimura A Changes in the HIV type 1 envelope gene from non-subtype B type-1 infected children in Kenya AIDS Res Hum Retroviruses 2009 25 2 141 148 19108688 10.1089/aid.2008.0144

[B21] Baeten JM Chohan B Lavreys I Chohan V McClelland RS Certain I Mandaliya K Jaoko W Overbaugh J HIV-1 sub-type D infection is associated with faster disease progression than sub-type A in spite of similar plasma HIV-1 loads J Infect Dis 2007 195 8 1177 1180 17357054 10.1086/512682

[B22] Sacktor N Nakasuija N Slolasky R Rezapour M Robertson K Musisi S Katabira E Ronald A Clifford D Laeyendecker O Quinn T HIV subtype D is associated with dementia compared with subtype A, in immunosuppressed individuals at risk of cognitive impairment in Kampala, Uganda Clin Infect Dis 2009 49 780 786 19622045 10.1086/605284 PMC2941149

[B23] Hunt PW Harrigan PR Huang W Bates M Williamson DW McCune JM Price RW Spudich SS Lampiris R Hoh T Leigler JN Deeks SG Prevalence of CXCR4 tropism among antiretroviral treated HIV-infected patients with detectable viremia J Infect Dis 2006 194 7 926 930 16960780 10.1086/507312

[B24] Deeks SG Martin JN Sinclair E Strong cell-mediated immune responses are associated with the maintenance of low-level viremia in antiretroviral-treated individuals with drug-resistant human immunodeficiency virus type 1 J Infect Dis 2004 189 312 321 14722897 10.1086/380098

[B25] Harouse JM Buckner C Gettie A CD8+ T cell-mediated CXC chemokine receptor 4-simian/human immunodeficiency virus suppression in dually infected rhesus macaques Proc Natl Acad Sci USA 2003 100 10977 10982 12963814 10.1073/pnas.1933268100 PMC196912

[B26] Andersson J Fehniger TE Patterson BK Early reduction of immune activation in lymphoid tissue following highly active HIV therapy AIDS 1998 12 123 129 10.1097/00002030-199811000-00004 9708402

[B27] Giovannetti A Ensoli F Mazzetta F CCR5 and CXCR4 chemokine receptor expression and beta-chemokine production during early T cell repopulation induced by highly active anti-retroviral therapy Clin Exp Immunol 1999 118 87 94 10540164 10.1046/j.1365-2249.1999.01033.x PMC1905399

[B28] Brumme ZL Goodrich J Mayer HB Molecular and clinical epidemiology of CXCR4-using HIV-1 in a large population of antiretroviral- naive individuals J Infect Dis 2005 192 466 474 15995960 10.1086/431519

[B29] Yuan W Craig S Si Z Farzan M Sodroski J CD4-induced T-20 binding to human immunodeficiency virus type 1 gp120 blocks interaction with the CXCR4 coreceptor J Virol 2004 78 5448 5457 15113923 10.1128/JVI.78.10.5448-5457.2004 PMC400340

[B30] Boucher CA Lange JM Miedema FF HIV-1 biological phenotype and the development of zidovudine resistance in relation to disease progression in asymptomatic individuals during treatment AIDS 1992 6 1259 1264 1282015 10.1097/00002030-199211000-00003

[B31] Low AJ Dong W Chan D Sing T Ronald S Jensen M Pillai S Good B Harrigan PR Current V3 genotyping algorithms are inadequate for predicting X4 co-receptor usage in clinical isolates AIDS 2007 21 14 17 24 10.1097/QAD.0b013e3282ef81ea 17721088

